# Psychogenic fever and neurodevelopmental disorders among Japanese children

**DOI:** 10.1186/s13030-024-00322-8

**Published:** 2024-12-18

**Authors:** Ayumi Okada, Yoshie Shigeyasu, Chikako Fujii, Chie Tanaka, Mana Hanzawa, Akiko Sugihara, Makiko Horiuchi, Hirokazu Tsukahara

**Affiliations:** 1 Department of Pediatrics, Faculty of Medicine, Dentistry and Pharmaceutical Sciences, Okayama University, 2-5-1 Shikata-cho, Kita-ku, Okayama, 700-8558 Japan; 2 Department of Pediatrics, Okayama University Hospital, 2-5-1 Shikata-cho, Kita-ku, Okayama, 700- 8558 Japan; 3 Department of Child Psychosomatic Medicine, Okayama University Hospital, 2-5-1, Shikata-Cho, Kita-Ku, Okayama, 700-8558 Japan; 4 Clinical Psychology Section, Department of Medical Support, Okayama University Hospital, 2-5-1, Shikata-Cho, Kita-Ku, Okayama, 700-8558 Japan

**Keywords:** Psychogenic fever, Functional hyperthermia, Neurodevelopmental disorder, Autism spectrum disorder, Environmental adaptation

## Abstract

**Background:**

Psychosocial stress can induce various physical symptoms, including fever, which is a commonly seen symptom in pediatric practice. In cases of unexplained fever, psychogenic fever should be considered as a potential cause. Children with neurodevelopmental disorders may be more vulnerable to stress and therefore more prone to developing somatic symptoms than their peers. This study aimed to elucidate the characteristics of children with psychogenic fever and comorbidity.

**Methods:**

This study included 21 patients with psychogenic fever who visited the Department of Pediatric Psychosomatic Medicine, Okayama University Hospital. Information on age, sex, disease onset, final estimated diagnosis, comorbidities, treatment course, and outcome was obtained from the patients’ medical records.

**Results:**

Of the 21 patients included, 7 were boys and 14 were girls, and their median age was 13.0 (range: 8.6–14.6) years. A total of 19 patients had no attendance at school, and all patients showed signs of maladjustment in school. The comorbidities included orthostatic dysregulation (*n* = 4) and migraine (*n* = 3). Neurodevelopmental disorders were observed in nine patients, eight of whom were diagnosed after the initial visit. The mean treatment duration was 37.2 months. The outcomes were complete remission (*n* = 9), improvement (*n* = 4), discontinuation (*n* = 1), and referral to another physician (*n* = 7).

**Conclusion:**

Various comorbidities were observed in the patients of this study with psychogenic fever, including the coexistence of neurodevelopmental disorders, such as autistic spectrum disorder. Children with neurodevelopmental disorders are prone to psychological stress resulting from difficulties in social adjustment. It is crucial to understand the developmental characteristics and environmental adaptation of patients to facilitate accurate diagnosis and treatment.

## Background

Psychogenic fever (PF) is characterized by elevated body temperature due to psychosocial stress. In pediatric practice, many patients present with a chief complaint of fever, and it is important to distinguish PF from fever of unknown origin (FUO). In some patients, psychosocial stress may be the cause of PF, but in children with difficulty verbalizing, the “psychological cause” may be unclear. PF is characterized by hyperthermia due to sympathetic hyperactivity rather than inflammation and, requires careful differentiation as no abnormalities are seen on examination. When parents and children worry about serious physical illnesses, there is a risk of doctor shopping. In a previous study, the only significant clinical parameter associated with doctor shopping was the presence of fever [1]. Furthermore, children spend most of their time in groups, e.g., at school, and the response to fever has become stricter due to the effects of COVID-19 countermeasures. Therefore, it is important to properly understand and manage PF.

Oka reported that a noninflammatory cytokine-mediated pathway exists for the mechanism of PF and that usual anti-inflammatory drugs are ineffective [2]. The underlying mechanisms are distinct from those of infection-induced fever and involve the central and sympathetic nervous systems. PF is characterized by a condition in which the core body temperature is high (up to 41 °C) or low-grade high (37–38 °C) during acute or chronic stress [3]. In one study of pediatric patients, PF accounted for 18% of all FUO cases and 2–6% of all psychosomatic disease cases [4]. In this population, PF is the common cause of FUO, and postural orthostatic tachycardia syndrome (POTS) is prevalent among children with PF. Enhanced sympathetic response to stress may play a pivotal role in both PF and POTS [5]. We previously reported 16 cases and, to the best of our knowledge, it was the first study to report on comorbidities associated with neurodevelopmental disorders [6]. The fact that patients have comorbidities may reflect some type of vulnerability among patients with PF. However, no studies have explored this point of view.

PF is a complex psychological, physiological, and endocrinological phenomenon [3]. In Japan, it is particularly observed during adolescence [4]. A better understanding of comorbidities will be beneficial for the pathogenesis and treatment of PF. This study aimed to examine the characteristics of PF in childhood, particularly the comorbidities, and the findings are expected to aid in the management of PF, for which no treatment has as yet been established.

## Subjects and methods

### Subjects

This study included 21 patients diagnosed with PF and treated at the Department of Pediatric Psychosomatic Medicine, Okayama University Hospital Pediatric Medical Center between April 1999 and March 2021. PF is a diagnosis of exclusion for which, no standardized diagnostic criteria have been established. It is characterized by the absence of an underlying organic disease with a certain period of non-fraudulent hyperthermia of 37.5 °C or higher occurring under acute or chronic psychosocial stress. It is defined as “a fever of 37.5°C” or higher that intermittently occurs over a period of 3 months or more in the absence of organic disease and in which psychosocial factors are thought to be involved. Patients with chronic fatigue syndrome and postinfectious hyperthermia were excluded.

### Methods

Information on age, sex, chief complaint, disease onset, whether the patient has been absent from school, final presumptive diagnosis, comorbidities, treatment course, and outcome was collected from the patients’ medical records. Orthostatic dysregulation (OD) was diagnosed based on symptoms and the use of active standing test according to the Orthostatic Regulation Japanese clinical guidelines [7]. However, for earlier cases, the diagnosis was based on the fulfillment of the OOKUNI criteria. Neurodevelopmental and psychiatric disorders were diagnosed using the Diagnostic and Statistical Manual of Mental Disorders, Fifth Edition, of the American Psychiatric Association [8]. Furthermore, previously diagnosed cases were reassessed using DSM-5, and a final diagnosis was made. To reach a diagnosis of neurodevelopmental disorder, a comprehensive assessment was conducted, including an interview regarding the patient’s upbringing, behavioral observation, intelligence testing, theory of mind testing, and collection of information from the school. Parent Interview ASD Rating Scale-Text Revision (PARS-TR) and Attention-Deficit/Hyperactivity Disorder Rating Scale (ADHD-RS) were also employed in some cases. The diagnosis was confirmed by three board-certified child mental health medical specialists in collaboration with the attending physician.

The outcomes were classified according to the presence or absence of fever symptoms and psychosocial disability: complete remission, asymptomatic and no interference with daily life; improved, asymptomatic but with interference in daily life; occasional symptoms, but interference in daily life has improved since the time of consultation; no change, symptoms and interference in daily life persist; worsened, symptoms have worsened and interference in daily life has increased; discontinued, treatment stopped; and transferred to another hospital, referred to another hospital because other symptoms were observed despite improvement of fever.

### Terminology

Functional hyperthermia (FH) is a condition characterized by high core body temperature without any inflammatory cause [2]. FH associated and induced with psychosocial stress has been diagnosed as PF. Children, in particular, experience elevated body temperatures in certain clinical situations; however, hyperthermia may persist in chronic stressful situations. In recent years, some studies have suggested that the term “functional hyperthermia” be used instead of “psychogenic fever” to avoid dualistic thinking, emphasize neural mechanisms, and separate complex cases from emotional hyperthermia in healthy subjects [2, 9]. In this study, FH complicated by psychological stress is called PF.

### Ethical considerations

This study was conducted in accordance with the protocol for “Study on the characteristics of pediatric patients with psychogenic fever” approved by the Ethics Committee of Okayama University Hospital (Research 2204-011).

## Results

### Patient characteristics

Of the 21 patients included, 7 were boys and 14 were girls (male/female ratio: 1:2), and their median age was 13.0 years (8 years 6 months to 14 years 9 months). (Table 1)

**Table 1:** Demographic and clinical characteristics of patients with PF

Characteristic	
Patients, n	21
Age (years), Median (low–high)	13.0 (8.6–14.9)
Sex, n	
Male	7
Female	14
School level, n	
Elementary school (8–12 years)	8
Junior high school (13–15 years)	13
No attendance at school, n (%)	19 (90.1)
Triggers and background of disease onset, n (including duplicates)	
School friendship problems	16
Academic failure	9
Parental illness, neglect	6
Sexual abuse	1
Comorbidities, n (including duplicates)	
Physical diseases	19
Psychological diseases	13
Neurodevelopmental disorders	9
Previously diagnosed with a neurodevelopmental disorder, n	
Yes	1
No	8
Recurrent fever during treatment, n(%)	9 (42.9)
Outcome, n	
End of remission	9
Improved	4
Discontinued	1
Transferred to another hospital	7
Period of the treatment duration (month), mean (SD)	37.2 (39.1)

Median (low–high) Mean(SD) SD: standard deviation

### Frequency

Approximately 1.7% of all outpatients examined during the 22 years covered in the study were affected.

### Family history

There were two patients who had siblings with neurodevelopmental disorders, one patient whose father had multiple sclerosis, and three patients whose mother or father had depression.

### Disease onset and background factors

There were overlapping cases. A total of 16 patients had interpersonal problems at school, such as bullying and friendship problems; 9 had learning difficulties (poor academic performance); 6 had family problems, such as parental illness and neglect; and 1 experienced sexual abuse.

### School maladjustment

Some patients had long absences from school and others attended school but frequently stayed in the nurse’s office: 19 (90.1%) patients had no attendance at school.

### Comorbid conditions (including overlapping conditions)

The comorbidities were neurodevelopmental disorders (*n* = 9), intellectual disability (ID) (*n* = 4), attention-deficit/hyperactivity disorder (*n* = 2), autism spectrum disorder (ASD) (*n* = 7), OD (*n* = 4), obesity (*n* = 4), migraine (*n* = 3), irritable bowel syndrome (IBS) (*n* = 2), bronchial asthma (*n* = 2), allergic rhinitis/atopic dermatitis (*n* = 1), and iron deficiency anemia (*n* = 1). Furthermore, there were 10 cases of adjustment disorder (AD) and 3 cases of social anxiety disorder (SAD). Among nine patients with comorbid neurodevelopmental disorders, four had comorbid AD (Table 2). The causes for these disorders were identified as bullying, poor friendships, and academic failure. The psychosocial factors of other patients were clearly psychological, including family and friendship problems and learning difficulties. Early improvement was observed in some patients who attended small group educational centers or took time off from school. However, some experienced recurrent fever when they attempted to return to school. In others, the condition persisted for several years. Only one out of nine patients had been diagnosed before the consultation, and the diagnosis was made based on the onset of PF.

**Table 2:** Characteristics of patients with comorbid neurodevelopmental disorders

ID	Sex	Age	Treatment duration	Onset	Comorbidities (final estimated diagnosis)			Intelligence quotient (WISC)					Outcomes
		(y, m)	(months)					FSIQ	VIQ	PIQ	WMI	PDI	
1	M	8.7	123	Bullying, transferred schools Poor academic performance	ID, ADHD	Obesity	SAD	74	82	76	73	83	Improved
2	M	10.2	1	Poor academic performance	ASD, ADHD	AR	-	77	90	80	57	94	End of remission
3	M	10.10	48	Problems with friends	ASD	-	AD	105	97	106	100	113	Improved
4	M	11.6	23	Problems with friends and family	ASD	-	AD	109	115	104	100	104	Improved
5	M	13.4	104	Problems with friends	ID, ASD	IBS	-	60	69	71	57	67	Transferred to another hospital
6	F	9.6	3	Poor academic performance	ID, ASD	-	AD	62	56	64	100	94	Transferred to another hospital
7	F	11.10	6	Unknown	ASD	OD	-	100	90	115	100	94	End of remission
8	F	12.10	61	Problems with friends	ASD	Migraine	AD	87	91	80	106	83	Improved
9	F	14.6	72	Poor academic performance Problems with friends	ID	Obesity BA	SAD	69	75	69			Transferred to another hospital

ID: intellectual disability, ADHD: attention-deficit/hyperactivity disorder, ASD: autism spectrum disorder, SAD: social anxiety disorder, AD: adjustment disorder

AR: allergic rhinitis, IBS: irritable bowel syndrome, OD: orthostatic dysregulation, BA: bronchial asthma

WISC: Wechsler Intelligence Scale for Children, FSIQ: full-scale intelligent quotient, VCI: verbal comprehension index, PRI: perceptual reasoning index, WMI: working memory index, PSI: processing speed index

### Treatment

All cases were treated as outpatients, except for case 5 who was admitted to the hospital for 4 days for a full investigation of FUO. All patients underwent lifestyle counseling (adjusting their daily rhythm, getting sufficient sleep and rest, providing guidelines for activity and rest when they have a fever, etc.) [6] and environmental adjustments (working with the school, standardizing their responses to fever, focusing on the mind–body connection, reducing stressors, and receiving counseling on ways to deal with the condition). In this study, it was difficult to evaluate the effectiveness of drug therapy because it was administered along with environmental adjustments and psychotherapy. One patient was given an antidepressant (paroxetine: Paxil^®^), which was ineffective, and eight patients were given an autonomic nervous system (ANS) regulator (tofisopam: Grandaxin^®^), which was effective in five patients (prolonged the period of normal temperature). Of the six patients who received a serotonin receptor antagonist (tandospirone: Sediel^®^), only three responded to the treatment. Herbal medicines, such as Hochuekkito, Shokenchuto, and Ninjinyoeito, were used, but only the latter showed effectiveness (increased the period of normal temperature). Because the drugs for PF were not covered by insurance, drug therapy was initiated after the parents and children were informed of the expected side effects, and their consent was obtained.

### Recurrence rate

Nine patients experienced fever recurrence during treatment due to environmental changes, such as schooling, new school term, or employment.

### Treatment duration

The mean treatment duration was 37.2 ± 39.1 months (standard deviation).

### Outcome

Of the 21 patients included, 9 went into complete remission, 4 improved, 1 discontinued treatment, and 7 were transferred to another hospital. Of the seven patients who were transferred to another hospital, two (cases 5 and 6) were diagnosed with ID and were referred to a hospital that could provide treatment. The fever of 2 other patients improved, but SAD was identified as a secondary disorder; thus, they were referred to a psychiatric department. The fever of the remaining three patients improved, but they stopped visiting the pediatric department when they entered high school and they were transferred to the internal medicine department for the treatment of physical illnesses (IBS, OD, and migraine). In addition, one patient who had bulimia nervosa and another (case 7) who had schizophrenia returned to the hospital after treatment completion.

## Discussion

PF is a stress-related psychosomatic illness that has been clinically recognized since the early 20th century. It commonly occurs in adolescents or young women and is characterized by acute or persistent elevation in body temperature [10]. A previous study in Japan examined 195 patients aged 3–56 years and reported that the peak age of onset was 13 years, with a slight prevalence in girls (male/female ratio of 1:1.19) [4]. In addition, it has been reported that 2.0% of the patients visited a psychosomatic medicine department with a chief complaint of fever, with a male/female ratio of 1:2.2 [11]. In our study, 1.7% of the patients visited the department, with a two-fold higher prevalence in girls than in boys. The low frequency is thought to be due to the fact that our hospital is a tertiary medical institution that is referred many cases of FUO after completing a detailed examination.

In this study, a high incidence of comorbid neurodevelopmental disorders was observed, particularly ASD. Children with neurodevelopmental disorders are prone to developing psychosomatic disorders. However, the coexistence of these disorders has not yet been reported. To the best of our knowledge, there have been no reports other than ours on the association between PF and neurodevelopmental disorders in children [6]. The hypothalamic–pituitary–adrenal (HPA) axis and ANS are neuroendocrine pathways associated with the stress system. There is substantial evidence indicating that children and adolescents with ASD exhibit atypical function within the HPA axis and ANS at rest and in the presence of social and/or nonsocial stressors [12]. Children with neurodevelopmental disorders are susceptible to psychosocial stressors as a result of their developmental characteristics and vulnerabilities and therefore tend to demonstrate maladaptive behaviors in school and other group settings. These children may have difficulty recognizing their feelings, expressing their emotions, and requesting assistance. Consequently, others may be unaware of their distress. In the present study, only one of the nine cases had been diagnosed before their visit to our hospital. The remaining eight cases were diagnosed in our clinic as a result of fever. Two potential explanations exist for the failure to diagnose the condition prior to the consultation. One potential explanation is that the diagnosis of a neurodevelopmental disorder is not solely based on the presence of specific developmental characteristics but also on the presence of a hindrance to daily life. DSM-5 [8] states that these symptoms may not become fully apparent until social demands exceed limited capacities or are masked by learned strategies in later life. Consequently, two of the nine patients had undergone an examination by a child psychiatrist, yet no definitive diagnosis was reached. An additional reason is that the evaluation of developmental characteristics may be delayed because of ruling out physical illness as a cause of fever. In children, a biopsychosocial approach is imperative for the treatment of psychosomatic disorders. In particular, it is essential to evaluate the developmental characteristics of patients when they present to the hospital with a chief complaint of FUO.

In cases with clear psychological causes, such as difficulty with certain classes, environmental adjustment are effective and improvement has been observed in a relatively short period of time. Furthermore, there has been cases in which symptoms improved with environmental changes, such as transferring schools, using an educational center, or continuing to higher education. The importance of environmental adjustment was once again made clear through the treatment of this disease. However, in cases with coexisting neurodevelopmental disorders, symptoms recurred due to increases in social stressors, such as work, even if fever improved. In addition, even if PF improved, there were cases in which treatment was continued due to social adaptation issues. Moreover, in this study, one case who had coexisting neurodevelopmental disorders was confirmed to have developed schizophrenia after the treatment. Patients with neurodevelopmental disorders are known to have various comorbidities. Specific patterns of comorbidity have important implications for etiology [13]. Another study found that the frequency of a history of psychiatric illness was higher in patients with FH than in those with other febrile illnesses [10]. There are several reports on the association between complaints of physical symptoms among children and the subsequent development of psychiatric symptoms and psychiatric disorders [14, 15]. A high prevalence of physical symptoms is a risk for subsequent mental health problems [16]. Youth with ASD were reported to have higher rates of epilepsy and allergies, but not asthma, compared with non-ASD youth [17]. However, there have been no reports on fever. It has been suggested that the effects of chronic stress are expressed in childhood as somatization in the form of fever, thereby affecting the vulnerability of individuals, and that as they grow up, this may lead to the onset of psychiatric illness in relation to environmental factors.

## Limitations

This study has several limitations. First, all patients were recruited from a single hospital that deals with referral patients. The patients included in this study were severely ill and may have significant psychosocial factors. Second, all patients were Japanese children, and the sample size was small. Thus, the generalizability of the findings is a concern. Finally, the present study did not clarify how the presence or absence of neurodevelopmental disorders affects the course of PF. Further research is warranted in the future.

## Conclusions

We herein assessed 21 patients with PF, of whom 9 (40.1%) had comorbid neurodevelopmental disorders. Our study demonstrated the characteristics of patients with PF suffering from chronic psychosocial problems and requiring long-term treatment. Environmental adjustment may be important in the treatment of these patients.

## Data Availability

Not applicable.

## Abbreviations

AD: Adjustment disorder
ADHD: Attention-deficit/hyperactivity disorder
AR: Allergic rhinitis
ASD: Autism spectrum disorder
BA: Bronchial asthma
CFS: Chronic fatigue syndrome
CRSD: Circadian rhythm sleep disorder
FUO: Fever of unknown origin
IBS: Irritable bowel syndrome
ID: Intellectual disability
OD: Orthostatic dysregulation
PF: Psychogenic fever
POTS: Postural orthostatic tachycardia syndrome
SAD: Social anxiety disorder
